# Hidradenitis suppurativa induced by ipilimumab and nivolumab: A rare association

**DOI:** 10.1016/j.jdcr.2024.12.009

**Published:** 2024-12-20

**Authors:** Alexander D. Woods, Katelyn Kim, Anna Axelson

**Affiliations:** a Department of Dermatology, University of Illinois College of Medicine at Chicago, Chicago, Illinois; b Department of Dermatology, Henry Ford Health System, Detroit, Michigan

**Keywords:** CTLA-4 inhibitors, cutaneous immune-related adverse events, hidradenitis suppurativa, immune checkpoint inhibitors, PD-1 inhibitors

## Introduction

Hidradenitis suppurativa (HS) is a debilitating chronic autoinflammatory disease of the hair follicle unit primarily affecting intertriginous areas of the body.^1^ HS often presents as multiheaded comedones, inflammatory nodules, and painful draining sinus tracts. Progression of the disease can result in extensive scarring.^1^

The etiology and pathogenesis of HS is complex and remains to be fully elucidated. It is thought to be multifactorial with infundibular hyperkeratosis of terminal follicles and hyperplasia of follicular epithelium leading to dilation and rupture of the folliculopilosebaceous unit, resulting in significant chronic inflammation.^1^^,^^2^ HS has been associated with smoking, obesity, and metabolic syndrome.^2^ In addition, HS may also be associated with other autoinflammatory syndromes such as Crohn’s disease, pyoderma gangrenosum, and spondyloarthropathies.^2^

Although tumor necrosis factor alpha inhibitors are commonly used to treat HS, there have been an increasing number of reports of HS induced by tumor necrosis factor alpha inhibitors.^3^^,^^4^ Other immunomodulatory agents have been reported to induce HS, including 2 reports of HS induced by an immune checkpoint inhibitor (ICI).^3^^,^^5^, ^6^, ^7^ We present a case of biopsy confirmed HS induced by ipilimumab and nivolumab.

## Case presentation

A 44-year-old African American obese male, former smoker, with metastatic renal cell carcinoma, was started on dual immunotherapy with ipilimumab and nivolumab for 4 cycles. Eleven days after the patient’s first cycle of dual immunotherapy, the patient was admitted to the hospital for a rash on his upper torso and extremities, gluteal abscess, and colitis. These were thought to be induced by the ICIs, especially ipilimumab-induced colitis, and resolved with cefepime, metronidazole, and cessation of ipilimumab. After admission, the patient was continued on nivolumab alone.

Two months later, the patient returned to the hospital with new perianal boils that developed 15 days after cycle 4. The patient presented to outpatient dermatology 4 months later for more widespread lesions. Examination revealed bilateral palpable subcutaneous nodules and tracts in the inguinal folds, scattered follicular-based papules in the axillae, groin, and gluteal cleft, and subcutaneous nodules with double-headed comedones in the beard distribution of his face and neck. The patient denied a history of previous HS lesions. The patient was diagnosed with nivolumab-induced HS, stage II. He was started on benzoyl peroxide 5% wash, clindamycin 1% solution twice daily, and doxycycline 100 mg twice daily.

**Fig 1:**
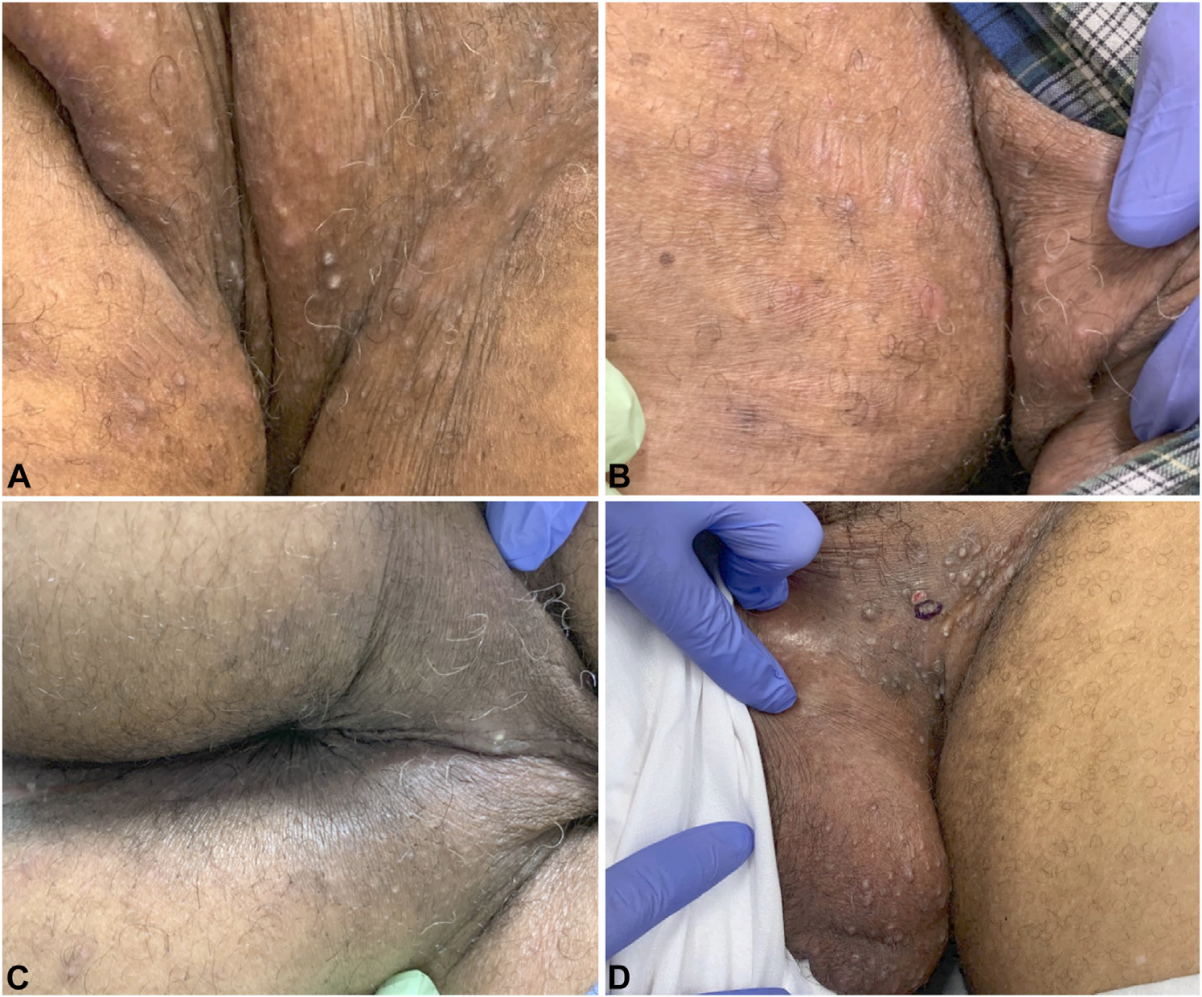
**A,** Perineum and inguinocrural folds with subcutaneous nodules and follicular-based papules; **B,** right inguinal fold and scrotum with subcutaneous nodules and follicular-based papules; **C,** perineum with subcutaneous nodules and follicular-based papules, healing site of prior incision and drainage in the left intergluteal cleft; and **D,** left inguinocrural fold and scrotum with subcutaneous nodules and follicular-based papules with biopsy site circled.

On 6-week follow-up, the patient noted some improvement in the face but unchanged activity in the groin, with new lesions every few days and painful flares occurring after every infusion. A few deep, palpable subcutaneous nodules were noted bilaterally in the groin and gluteal region (Fig 1). He had self-discontinued therapy after 4 weeks; thus, he was restarted on benzoyl peroxide, topical clindamycin, and doxycycline for 3 months.

**Fig 2:**
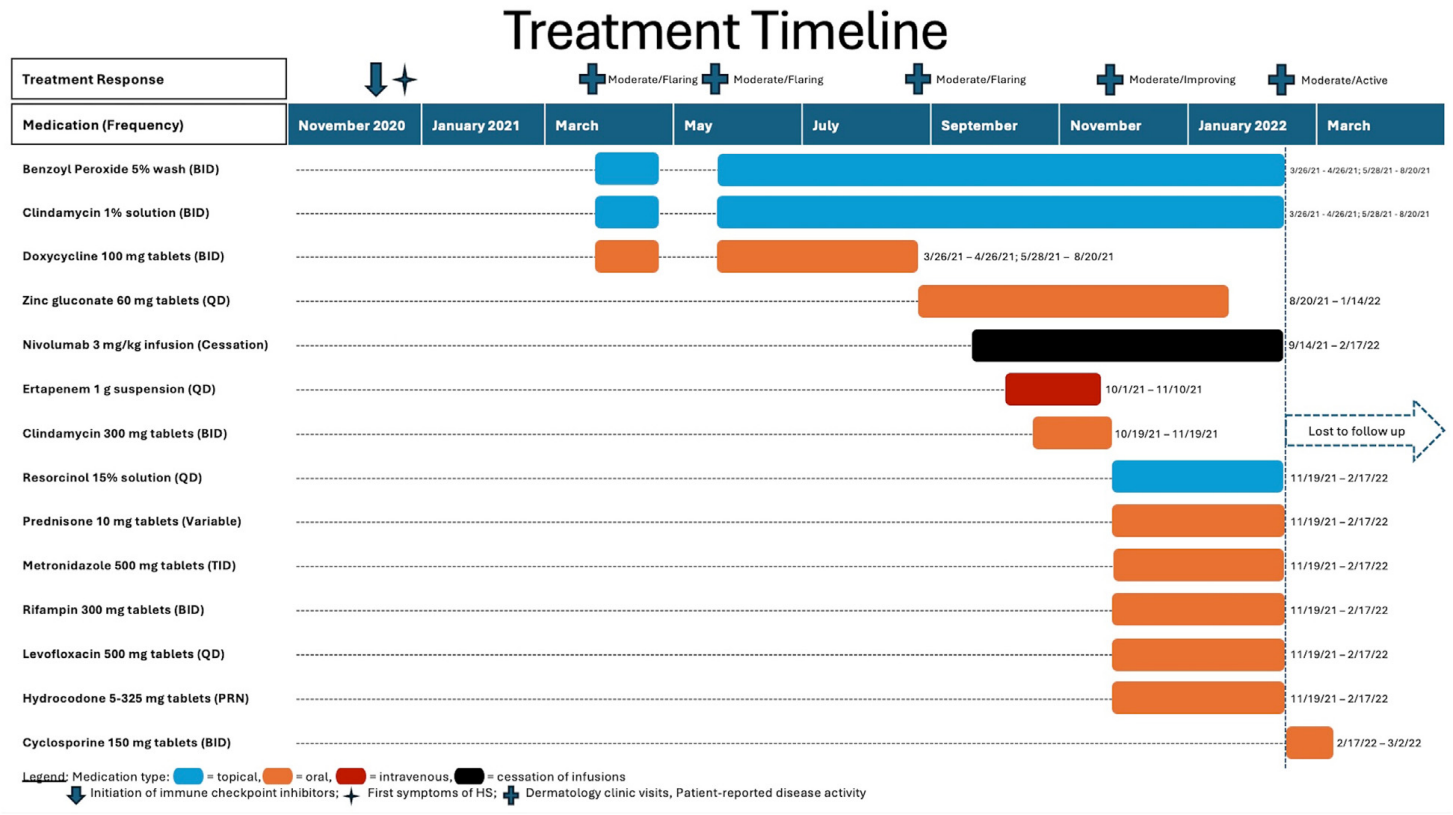
Treatment timeline and patient-reported disease severity.

The patient continued experiencing painful lesions despite treatment. Punch biopsy of the left inguinal fold revealed perifolliculitis, with follicular plugging and dermal fibrosis, consistent with HS. Bacterial culture grew many *Corynebacterium simulans*. Numerous topical and systemic treatment regimens were trialed with poor to mild improvement in symptoms (Fig 2). Repeat incisional biopsies were obtained to rule out cutaneous metastases due to the recalcitrant nonhealing nature of the nodules and were again consistent with HS. Ultimately, because of cancer disease stability, the decision was made to discontinue nivolumab infusions. Although he continued to have ongoing disease, this proved to reduce his symptoms the most, with gradual improvement and eventually only mild intermittent flares. The patient was subsequently lost to follow-up.

## Discussion

Targeted biologic immune-modulating agents and ICIs have revolutionized the care of rheumatologic disorders and provided new approaches for cancer treatment, respectively. However, numerous cutaneous immune-related adverse events (cirAEs) have been reported with these therapies. This case highlights the induction of HS from the combination of the ICIs ipilimumab, a cytotoxic T-lymphocyte-associated protein-4 inhibitor, and nivolumab, a programmed cell death protein-1 (PD-1) receptor inhibitor.

Although the patient has multiple HS risk factors, the temporal association of ICI treatments, new onset of HS symptoms, histopathologic findings, and improvement of symptoms after ICI cessation support ICI-induced HS diagnosis. This case is important as it builds off 2 previous reports of HS induced by ICIs.^6^^,^^7^ These cases presented with classic morphologic features of HS. Although our patient initially demonstrated draining abscesses and painful nodules, their disease evolved to later predominantly exhibit folliculocentric papules and papulonodules. Of note, all 3 cases demonstrated poor response to tetracycline antibiotics, and our patient had a suboptimal response to ertapenem. This may be due to bacterial activation of the immune system playing a smaller role in the disease state in ICI-induced HS and the more papular morphology for our patient.

HS is a chronic condition with a debilitating course. Although rare, early recognition and treatment of HS in patients using ICIs and other biologic agents may mitigate some of the morbidity associated with HS. Although the mechanism by which ICIs induce HS is not fully understood, it is proposed that the disinhibition of T-cell regulation leads to nonspecific T-cell activation that may increase T helper 17 cells and interleukin 17 and recruit neutrophils in the skin. This may be responsible for unmasking HS in susceptible individuals as interleukin 17 is increased in perilesional and lesional HS skin.^6^

Anti-PD-1 treatments can result in cirAEs in approximately 40% of patients.^8^ These cirAEs may present weeks to years after initiation of anti-PD-1 therapies and may even present after discontinuation of therapy.^8^ Occurrence of cirAEs may additionally predict increased antineoplastic efficacy of ICIs with increased overall survival and progression-free survival.^9^^,^^10^ Further studies are needed to identify if ICI-induced HS is a positive prognostic factor in cancer patients receiving ICIs. Prior reports suggest drug-induced HS may respond to discontinuation of the inciting agent. This was true for Maillard et al^6^ and our patients, who experienced the most benefit from nivolumab cessation.^3^ Early identification and treatment of drug-induced HS may prevent disfigurement, decrease morbidity, and improve patient quality of life. Treatment may necessitate systemic therapy, and although biologics such as tumor necrosis factor alpha inhibitors are contraindicated in patients with cancer, interleukin 17 inhibitors (eg, secukinumab) may offer a promising treatment strategy for these patients. Unfortunately, secukinumab was not approved yet for HS during this patient’s treatment. Classic antibiotic treatments for HS may prove less effective in ICI-induced HS because of the primarily ICI-driven nature, however, further research is needed to clarify the best treatment approach.

## Conclusion

This case highlights the association of HS with the combination of PD-1 and cytotoxic T-lymphocyte-associated protein-4 inhibitor ICIs. Although rare, knowledge of this association can aid in the early recognition and treatment of cirAEs. Furthermore, recognition of cirAEs, such as HS, may be a useful prognostic factor of therapeutic response to ICIs in patients with cancer. Additional studies are needed to further elucidate whether flares of HS consistently correspond to tumor response and the impact of ICI cessation on disease course.

## Conflicts of interest

None disclosed.
